# Phosphorous Application Improves Drought Tolerance of *Phoebe zhennan*

**DOI:** 10.3389/fpls.2017.01561

**Published:** 2017-09-13

**Authors:** Akash Tariq, Kaiwen Pan, Olusanya A. Olatunji, Corina Graciano, Zilong Li, Feng Sun, Xiaoming Sun, Dagang Song, Wenkai Chen, Aiping Zhang, Xiaogang Wu, Lin Zhang, Deng Mingrui, Qinli Xiong, Chenggang Liu

**Affiliations:** ^1^CAS Key Laboratory of Mountain Ecological Restoration and Bioresource Utilization and Ecological Restoration Biodiversity Conservation Key Laboratory of Sichuan Province, Chengdu Institute of Biology, Chinese Academy of Sciences Chengdu, China; ^2^International College, University of Chinese Academy of Sciences Beijing, China; ^3^Instituto de Fisiología Vegetal, Consejo Nacional de Investigaciones Científicas y Técnicas – Universidad Nacional de La Plata Buenos Aires, Argentina; ^4^Key Laboratory of Tropical Plant Resources and Sustainable Use, Xishuangbanna Tropical Botanical Garden, Chinese Academy of Sciences Chengdu, China

**Keywords:** *Phoebe zhennan*, drought stress, phosphorous fertilization, drought tolerance, conservation

## Abstract

*Phoebe zhennan* (Gold Phoebe) is a threatened tree species in China and a valuable and important source of wood and bioactive compounds used in medicine. Apart from anthropogenic disturbances, several biotic constraints currently restrict its growth and development. However, little attention has been given to building adaptive strategies for its conservation by examining its morphological and physio-biochemical responses to drought stress, and the role of fertilizers on these responses. A randomized experimental design was used to investigate the effects of two levels of irrigation (well-watered and drought-stressed) and phosphorous (P) fertilization treatment (with and without P) to assess the morphological and physio-biochemical responses of *P. zhennan* seedlings to drought stress. In addition, we evaluated whether P application could mitigate the negative impacts of drought on plant growth and metabolism. Drought stress had a significant negative effect on the growth and metabolic processes of *P. zhennan*. Despite this, reduced leaf area, limited stomatal conductance, reduced transpiration rate, increased water use efficiency, enhanced antioxidant enzymes activities, and osmolytes accumulation suggested that the species has good adaptive strategies for tolerating drought stress. Application of P had a significant positive effect on root biomass, signifying its improved water extracting capacity from the soil. Moreover, P fertilization significantly increased leaf relative water content, net photosynthetic rate, and maximal quantum efficiency of PSII under drought stress conditions. This may be attributable to several factors, such as enhanced root biomass, decreased malondialdehyde content, and the up-regulation of chloroplast pigments, osmolytes, and nitrogenous compounds. However, P application had only a slight or negligible effect on the growth and metabolism of well-watered plants. In conclusion, *P. zhennan* has a strong capability for drought resistance, while P application facilitates and improves drought tolerance mostly through physio-biochemical adjustments, regardless of water availability. It is imperative to explore the underlying metabolic mechanisms and effects of different levels of P fertilization on *P. zhennan* under drought conditions in order to design appropriate conservation and management strategies for this species, which is at risk of extinction.

## Introduction

Scenarios of climatic change predict an increase in the duration and severity of the drought events in major parts of the world, which will potentially affect nutrient availability, plant growth and productivity, and ecosystem function (Ledger et al., 2013; He and Dijkstra, 2014). The increased production of reactive oxygen species (ROS), as a result of drought stress, induces a series of morphological and metabolic changes that affect normal growth and development of many plant species (Keunen et al., 2013; Oliveira et al., 2014). Stress signals are first perceived at the membrane level by the receptors and then transduced in the cell to up-regulate stress response genes that facilitate stress tolerance (Mahajan and Tuteja, 2005). However, tolerance to environmental stress varies depending on plant species, growth stage and stress intensity (Demirevska et al., 2009).

Phosphorus (P) is a key element required for normal plant development, but its low mobility in soil results in poor uptake by plants, which consequently inhibits growth and metabolism. The majority of soil types, including fertile soils, have low available P, because the rate of absorption in the rhizosphere exceeds the rate of its replenishment in soil solution (Suriyagoda et al., 2011). Previous studies suggest that phosphorus contributes for the extension of root system and P deficiency will exacerbate drought stress (Cramer et al., 2009; Sardans and Penuelas, 2012). The use of P fertilizer reduces its deficiency in soil, increases the stress-tolerating ability of plants (Cortina et al., 2013) and results in adjustments of physiological, morphological, and biochemical processes that increase plant growth (dos Santos et al., 2004; Jones et al., 2005; Campbell and Sage, 2006; Faustino et al., 2013; Liu et al., 2015). Despite the importance of P in plant productivity, relatively few studies have assessed its effects on plant physiological and ecological processes under drought stress (dos Santos et al., 2006; Naeem and Khan, 2009; Fleisher et al., 2012; Jin et al., 2015; Liu et al., 2015). Most of these studies examined the relationship between P application and physio-biochemical adjustments under drought stress in non-woody plants (mostly crop species). Much less attention has been paid to evaluating the relationship between P application and drought resistance mechanisms in high quality, timber producing forest trees of conservation interest; this could be a potential area of research due to issues of possible future climate change.

Forest trees account for approximately 82% of terrestrial biomass and more than 50% of terrestrial biodiversity (Neale and Kremer, 2011), they help mitigate against climate change and provide a range of ecosystem services, such as nutrient cycling, carbon sequestration, water and air purification, and timber production for energy and industry (Harfouche et al., 2014). Globally, forest trees are increasingly subject to different types of environmental stresses that affect the growth and sustainability of trees. The predicted increase in global drought stress is likely to significantly impact forest trees by reducing nutrient supply and uptake and altering its redistribution in soils through mineralization (Schimel et al., 2007; Sanaullah et al., 2012). Studies on the effects of nutrient availability on the growth of woody species are common (He and Dijkstra, 2014), but there is little understanding about the possible role of nutrient application in the enhancement of stress tolerance ability of forest tree species.

The slow-growing, forest tree species, *Phoebe zhennan* S. Lee (Lauraceae; Golden Phoebe), is endemic to subtropical China and is widely distributed at elevations below 1,500 m (Hu et al., 2015). While *P. zhennan* is an economically important timber and medicinal species, it has been listed as a threatened species by International Union for Conservation of Nature (IUCN) and is nationally protected (Gao et al., 2016). Apart from anthropogenic disturbances, studies of forest plantations have suggested that *P. zhennan* rarely reaches its full growth potential due to abiotic constraints (Wu, 2009) such as drought stress. Although Hu et al. (2015) studied the antioxidative response of *P. zhennan* to drought conditions; this was a short-term study of 1 month duration and, as such, does not provide a detailed understanding of the effect of drought stress on growth and physio-biochemical impairment or adaptive strategies. Nevertheless, it has been suggested that slow-growing species are less sensitive to soil mineral and drought stress, because of their low mineral absorption rate and slow growth strategy (Chapin, 1980) and it is possible that *P. zhennan* may possess inherent strategies to abiotic stress. There is a lack of understanding of growth and metabolic responses by *P. zhennan* to drought stress and the possible associated morphological and physio-biochemical adaptive strategies. Moreover, to the best of our knowledge, there is no published research examining how P fertilizer application may help mitigate drought stress in *P. zhennan*. The likelihood of climate change mediated increases in arid conditions may have implications for the economically important, but threatened *P. zhennan*. Thus, research to promote the understanding of drought tolerance and response mechanisms to addition of P fertilizer in *P. zhennan* is timely.

In this study, we addressed the following objectives (1) to assess the morphological and physio-biochemical responses of *P. zhennan* to drought stress; and (2) to evaluate whether P application mitigates the negative impact of drought by improving the tolerance capacity of *P. zhennan*. Accordingly, we investigated plant growth, water status, gas exchange, chlorophyll fluorescence, reactive oxygen production rate, antioxidant enzyme activities, and biochemical parameters.

## Materials and Methods

### Experiment Design

The experiment was conducted at the Center for Ecological Studies at the Chinese Academy of Sciences, Sichuan in south-west China. Healthy and uniform, 2-year-old *P. zhennan* plants were collected from Sichuan Agricultural University, Sichuan province, and transplanted to 10 L plastic pots filled with approximately 4 kg of homogenized topsoil (pH 7.3, total nitrogen 0.19%, and carbon 2.67%). The pots were arranged in a complete randomized block design in a greenhouse (temperature range 18–32°C, relative humidity range 50–85%) and regularly watered. Light availability was homogeneous inside the greenhouse, and direct sunlight reduction due to covering was in the range of 6–9%. After growing for 2 months in the greenhouse, the plants were subject to three replicates of four treatments for 90 days: two water regimes (well-watered and water-stressed) and two levels of P fertilization (with and without P fertilization). Immediately prior to applying the treatments, total and available P in soils was first determined to be 0.89 g kg^-1^ and 27.6 mg kg^-1^, respectively. Available P was extracted with 0.5 M NaHCO_3_ (pH 8.2) according to Olsen and Sommers (1982) and measured colorimetrically by the molybdate-ascorbic acid as described by Murphy and Riley (1962). Soil relative water content (SRWC) of the two water treatments (control: 80–85%; severe drought: 30–35%) was calculated using the weight method (Xu et al., 2009). The pots were weighed daily and watered up to their respective target SRWC, by replacing the amount of water transpired and evaporated. SRWC was expressed as:

$$\text{SRWC=}(W_{soil}+W_{pot}+{DW}_{soil})/\left(W_{FC}-W_{pot}\right)$$

where *W*_soil_ was the current soil weight, *W*_pot_ was the weight of the empty pot, *DW*_soil_ was the dry soil weight, and *W*_FC_ was the soil weight at field capacity.

Phosphorous fertilization was supplied as sodium dihydrogen phosphate (NaH_2_PO_4_, 25.5% P) with the dose of 129.3 mg P mixed in 200 mL water per pot every 30 days. In order to avoid systematic error produced by fluctuation in environmental conditions, pots were rotated after every 5 days during the experiment. Each treatment was replicated three times. Plant samples were collected at the end of the experiment.

### Plant Growth and Biomass

Plant height (cm), stem diameter (mm), and leaf area (cm^2^) were measured by using a measuring tape, caliper, and a leaf area meter (CI 202, United States), respectively. After removing the plants from the soil, roots, stems, and leaves were separated and weighed. Samples were oven dried at 70°C for 24 h, to measure biomass.

### Leaf Relative Water Content

Expanded leaves were collected from each plant and weighed to obtain fresh weight (FW). The leaves were then immediately dipped into distilled water at a temperature of 4°C and under dark conditions. After 12 h, leaves were weighed to obtain turgor weight (TW) and then dried for 24 h in an oven set at 70°C to determine dry weight (DW). The following equation was used to calculate leaves relative water content (LRWC).

LRWC=[(FW−DW)/(TW−DW)]×100%.

### Gas Exchange and Chlorophyll Fluorescence

The net CO_2_ assimilation rate (*P*_n_), stomatal conductance (*G*_s_), and intercellular CO_2_ concentration (*C*_i_) were measured with a portable open-flow gas exchange system (LI-6400, LI-COR Inc., United States), between 9:00 and 11:00 h, on fully expanded leaves that were at similar stages of development. During this time, relative air humidity, CO_2_ concentration, and photon flux density were maintained at 60–70%, 380 μmol mol^-1^ and 800 μmol m^-2^ s^-1^, respectively. Intrinsic water use efficiency (WUE_intr_) was calculated by dividing the instantaneous values of *P*_n_ by *G*_s_. The maximum quantum efficiency of photosystem II (*F*_v_*/F*_m_) of the leaves was measured with a portable pulse amplitude modulated fluorometer (PAM-2100, Walz, Effeltrich, Germany), where the leaves were dark-adapted with clips for 20 min. After this time, minimal fluorescence (*F*_o_) was measured under a weak pulse of modulating light over 0.8 s, and maximal fluorescence (*F*_m_) was induced by a saturating pulse of light (8,000 mmol m^-2^ s^-1^) applied over 0.8 s. *F*_v_*/F*_m_ was calculated, where *F*_v_ was the difference between *F*_m_ and *F*_o_.

### Photosynthetic Pigments

Chlorophyll *a* (Chl *a*), chlorophyll *b* (Chl *b*), and carotenoids (Car) were determined using 0.2 g FW leaf samples, with 80% acetone as a solvent. Leaf samples were placed in dark conditions for 36 h and absorbance was recorded at 662, 645, and 470 nm spectrophotometrically (Lichtenthaler and Buschmann, 2001).

### Determination of Biochemical Parameters

Dried leaf samples (0.2 g DW) were mixed with 6 mL of 80% ethanol at 80°C for 30 min. The resulting extracted supernatant was analyzed for soluble sugars (SS) following the anthrone method using sucrose as a standard (Zhang and Qu, 2003). For NO_3_^-^ concentration, 0.2 g of frozen leaves was homogenized in 5 mL of deionized water, while for NH_4_^+^ concentration, 0.2 g of frozen leaves was homogenized in 2 mL of 10% HCl. The resulting supernatants were analyzed using a quantitative colorimetric method as described by Tang (1999). Proline was extracted with 2 mL of 10% acetic acid and 5 mL of 3% sulfosalicylic acid, respectively. The resulting supernatants were analyzed according to the method of Liu et al. (2014). Soluble proteins (SP) were determined using Bradford G-250 reagent.

### ROS and Lipid Peroxidation

Two measures of ROS were estimated. Firstly, production rate of superoxide anion (O_2_^⋅-^) was measured by monitoring nitrite formation from hydroxylamine, in the presence of O_2_^⋅-^ (Elstner and Heupel, 1976). Fresh leaves (0.2 g) were homogenized with 2 mL of 65 mM phosphate buffer (pH 7.8) and centrifuged at 5000 × *g* for 10 min. The incubation mixture contained 0.9 mL of 65 mM phosphate buffer (pH 7.8), 0.1 mL of 10 mM hydroxylammonium chloride, and 1 mL of supernatant. After incubation at 25°C for 20 min, 17 mM sulphanilamide, and 7 mM α-naphthylamine were added to the incubation mixture and kept at 25°C for 20 min. Ethyl ether in the same volume was added and centrifuged at 1500 × *g* for 5 min. The absorbance wavelength for the aqueous solution was 530 nm.

Secondly, hydrogen peroxide (H_2_O_2_) concentration was determined by monitoring the absorbance of the titanium-peroxide complex at 410 nm (Patterson et al., 1984). Fresh leaves (0.2 g) were homogenized with 5 mL of acetone and centrifuged at 3,000 × *g* for 10 min. The reactive mixture, containing 0.1 mL of titanium reagent (50 μL of 20% titanium tetrachloride in concentrated HCl), 0.2 mL of ammonia, and 1 mL of supernatant, was centrifuged at 3,000 × *g* for 10 min. The resulting precipitate was washed five times with acetone, before being centrifuged at 10,000 × *g* for 5 min. The precipitate was solubilized in 3 mL of 1 M H_2_SO_4_ and the absorbance at 410 nm was measured. Lipid peroxidation was estimated by measuring malondialdehyde (MDA) content according to the thiobarbituric acid (TBA) test at 450, 532, and 600 nm, respectively (Zhou et al., 2007). For MDA assay, 0.25 g of fresh leaves were ground in 5 mL of 1% trichloroacetic acid (TCA) and centrifuged at 5,000 *g* for 10 min. Supernatant (1 mL) was added to 4 mL of 20% TCA (containing 0.5% TBA) and the mixture was heated at 95°C for 30 min, before being cooled in an ice bath. Absorbance was read using a spectrophotometer at 450, 532, and 600 nm (Zhou et al., 2007). The MDA concentration was calculated using the following equation:

$$MDA(molg^{-1}FW)=6.45(OD532-OD600)-0.56OD450$$

### Antioxidant Enzyme Activities

Three measures of antioxidant stress were assessed. Superoxide dismutase (SOD) activity was determined using the nitroblue tetrazolium (NBT) method (Fu and Huang, 2001). One unit of SOD activity was defined as the amount of enzyme required for 50% inhibition of NBT reduction at 560 nm. Activities of catalase (CAT) and peroxidase (POD) were determined using the methods of Fu and Huang (2001). For CAT, the decomposition of H_2_O_2_ was determined by measuring the reduction in absorbance at 240 nm for 1 min. For POD, the oxidation of guaiacol was determined by measuring the increase in absorbance at 470 nm for 1 min. One unit of CAT and POD activity was defined as an absorbance change of 0.01 unit’s min^-1^.

### Statistical Analysis

All measurements were repeated three times, and the data were organized using Microsoft Excel 2007 and presented as means ± standard errors (SEs). SPSS version 16.0 (Chicago, IL, United States) was used to run one-way analysis of variance (ANOVA) and Duncan’s multiple range tests at the 0.05 significance probability level were used to compare mean values. Prior to analysis, data were checked for normality and homogeneity of variances. Origin pro 8.5 was used for graphical presentation; error bars represent standard errors, and all data in the figures represent the means ± SEs.

## Results

### Plant Growth and Biomass

Reduction in morphological traits was observed in drought-stressed plants compared with well-watered plants (**Table 1**). Under water deficit conditions, shoots and root biomass, leaf area and stem diameter significantly decreased (45.2, 39.8, 28.5, and 9.1%, respectively) compared with well-watered plants irrespective of P application. Root biomass in drought-stressed plants was significantly higher (14.4%) in fertilized plants than in unfertilized plants. There were no significant treatment differences for the other growth parameters.

**Table 1:** Changes in morphological parameters of *Phoebe zhennan* for non-fertilized (-P) and fertilized (+P) treatments with and without water stress.

Traits	Well-watered		Water-stressed	
		
	-P	+P	-P	+P
Leaf biomass (g)	2.12 ± 0.29a	1.98 ± 0.48a	1.52 ± 0.08a	1.55 ± 0.06a
Shoot biomass (g)	9.99 ± 0.9a	9.7 ± 0.87a	5.47 ± 0.42b	5.96 ± 0.45b
Root biomass (g)	5.18 ± 0.17a	5.37 ± 0.12a	3.12 ± 0.08c	3.57 ± 0.12b
Leaf area (cm^2^)	32.55 ± 3.03a	32.72 ± 1.73a	23.26 ± 1.1b	22.19 ± 0.71b
Height (cm)	34.33 ± 1.2a	35.33 ± 2.19a	32.33 ± 1.2a	33.33 ± 0.88a
Diameter (mm)	4.81 ± 0.1a	4.68 ± 0.11ab	4.37 ± 0.09b	4.47 ± 0.13ab


*Means followed by different letters indicate significant differences (*P* ≤ 0.05) among the four treatments according to Duncan’s test. Values are means ± SE.*

### Leaf Relative Water Content, Photosynthetic and Chlorophyll Fluorescence

In comparison with well-watered unfertilized plants there was a significant reduction in LRWC, *P*_n_, *F*_v_*/F*_m_, *C*_i_, and *G*_s_ (27.2, 72.6, 17.2, 34.72, and 73.94%, respectively) of drought-stressed unfertilized plants. Under drought stress conditions, LRWC, *P*_n_, and *F*_v_*_/_F*_m_ were significantly lower in unfertilized plants than in fertilized plants, while there were no significant effects of fertilizer on the other parameters in drought-stressed plants (**Table 2**). Water use efficiency (WUE_intr_) showed an opposite trend and increased 44.13% under drought condition than well-watered plants, regardless of P application. However, there was no significant change in WUE_intr_ in P fertilized plants under water-stressed conditions. There was no effect of P fertilizer on LRWC or any of the photosynthetic and chlorophyll parameters in well-watered plants. Moreover, under drought condition water consumption rate was higher in P-fertilized plants than unfertilized plants but lower than well-watered plants (data not shown).

**Table 2:** Changes in leaf relative water content, photosynthetic and chlorophyll fluorescence parameters, water use efficiency in *P. zhennan* for non-fertilized (-P) and fertilized (+P) treatments with and without water stress.

Traits	Well-watered		Water-stressed	
		
	-P	+P	-P	+P
LRWC (%)	41.5 ± 1.93a	41.7 ± 2.74a	30.2 ± 0.89b	36.3 ± 1.16a
*P*_n_ (μmol m^-2^ s^-1^)	2.57 ± 0.11ab	3.11 ± 0.3a	1.24 ± 0.07c	2.03 ± 0.29b
*C*_i_ (μmol mol^-1^)	248.21 ± 17.16a	249.91 ± 15.8a	162.02 ± 10.8b	207.88 ± 16.39ab
*G*_s_ (mol m^-2^ s^-1^)	0.036 ± 0.004ab	0.047 ± 0.0136a	0.0093 ± 0.0003c	0.0209 ± 0.0049bc
WUE_intr_ (μmol mol^-1^)	74.4 ± 11.73b	73.17 ± 12.53b	133.18 ± 7.17a	101.51 ± 11.16ab
*E* (mmol m^-2^ s^-1^)	1.51 ± 0.18ab	1.86 ± 0.62a	0.4 ± 0.01b	0.89 ± 0.17ab
*F*_v_/*F*_m_	0.81 ± 0.02a	0.82 ± 0.02a	0.67 ± 0.01c	0.74 ± 0.02b


*Means followed by different letters indicate significant differences (*P* ≤ 0.05) among the four treatments according to Duncan’s test. Values are means ± SE.*

### Photosynthetic Pigments

**FIGURE 1:**
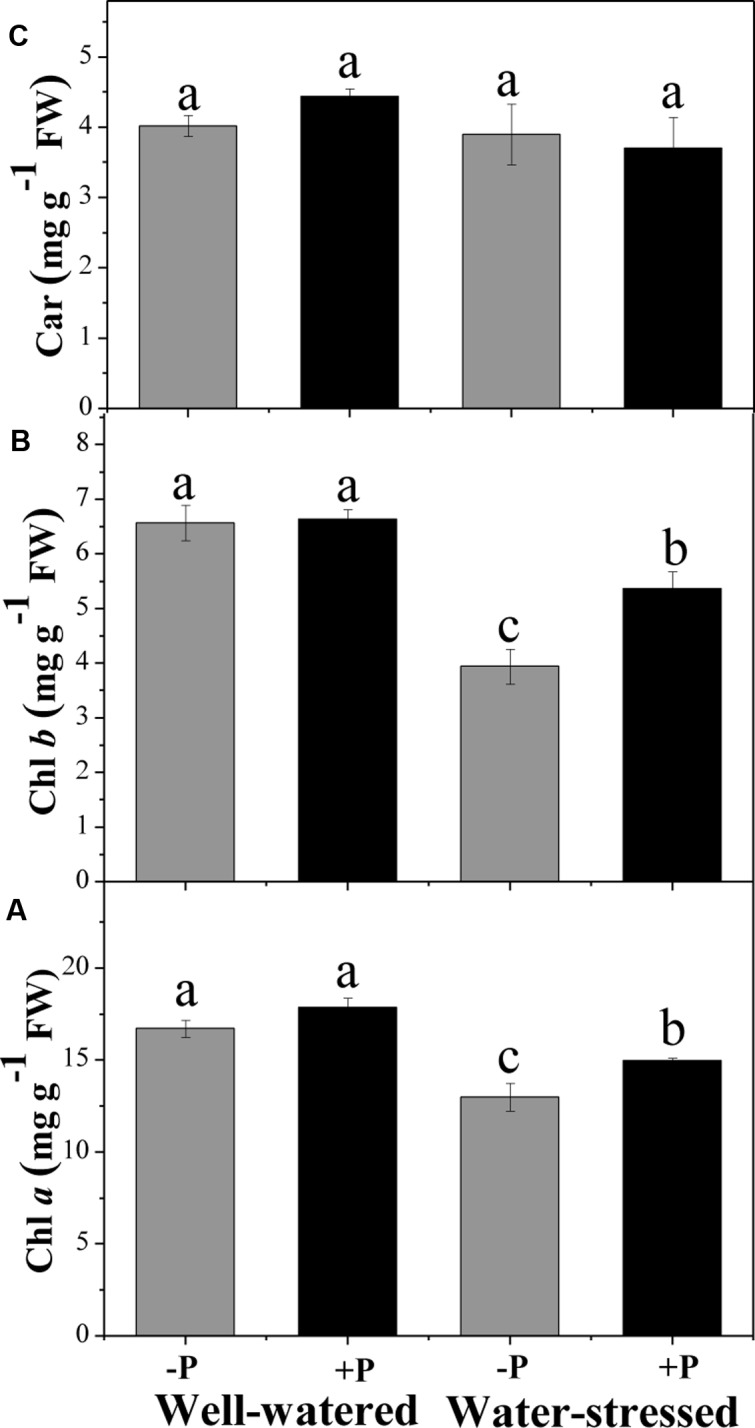
Changes in chloroplast pigments, chlorophyll a (Chl *a*, **A**), chlorophyll b (Chl *b*, **B**), and carotenoids (Car, **C**) for fertilized (+P) and non-fertilized (–P) treatments with and without water. Means followed by different letters indicate significant differences (*P* ≤ 0.05) among the four treatments according to Duncan’s test. Vertical bars show ± SE.

Concentration of Chl *a* and Chl *b* in non-fertilized plants was significantly lower (22.2 and 40.0%, respectively) in water-stressed than in well-watered plants (**Figures 1A,B**). We found that Chl *a* and Chl *b* concentration was significantly greater in drought-stressed plants that had been treated with fertilizer than in those that had been unfertilized. Neither water nor fertilizer treatment had any effect on carotenoid concentration (**Figure 1C**).

### Biochemical Parameters

The concentration of NH_4_^+^ was higher in well-watered plants than drought-stressed plants, while the opposite was found for proline concentration (**Table 3**). With the exception of SS concentration, which was greater in fertilized than unfertilized drought-stressed plants, we found no significant effect on biochemical parameters of fertilizer within well-watered or drought-stressed plants.

**Table 3:** Osmolytes accumulation (soluble sugars concentration) and concentration of nitrogenous compounds reduction and assimilation in *P. zhennan* for non-fertilized (**-**P) and fertilized (+P) treatments with and without water stress.

Traits	Well-watered		Water-stressed	
		
	-P	+P	-P	+P
Soluble sugars (mg g**^-^**^1^ DW)	0.44 ± 0.01b	0.41 ± 0.02b	0.47 ± 0.02b	0.57 ± 0.03a
NH_4_^+^ (mg g**^-^**^1^ DW)	0.93 ± 0.03a	0.93 ± 0.06a	0.63 ± 0.04b	0.77 ± 0.058b
NO_3_**^-^** (mg g**^-^**^1^ DW)	2.75 ± 0.2a	2.85 ± 0.1a	2.01 ± 0.16b	2.33 ± 0.12ab
Soluble proteins (mg g**^-^**^1^ DW)	65.33 ± 4.03ab	67.55 ± 1.6a	57.94 ± 0.9b	60.1 ± 1.41ab
Proline (mg g**^-^**^1^ DW)	0.037 ± 0.001b	0.036 ± 0.003b	0.055 ± 0.006a	0.057 ± 0.004a


*Means followed by different letters indicate significant differences (*P* ≤ 0.05) among the four treatments according to Duncan’s test. Values are means ± SE.*

### ROS Production and Lipid Peroxidation

**FIGURE 2:**
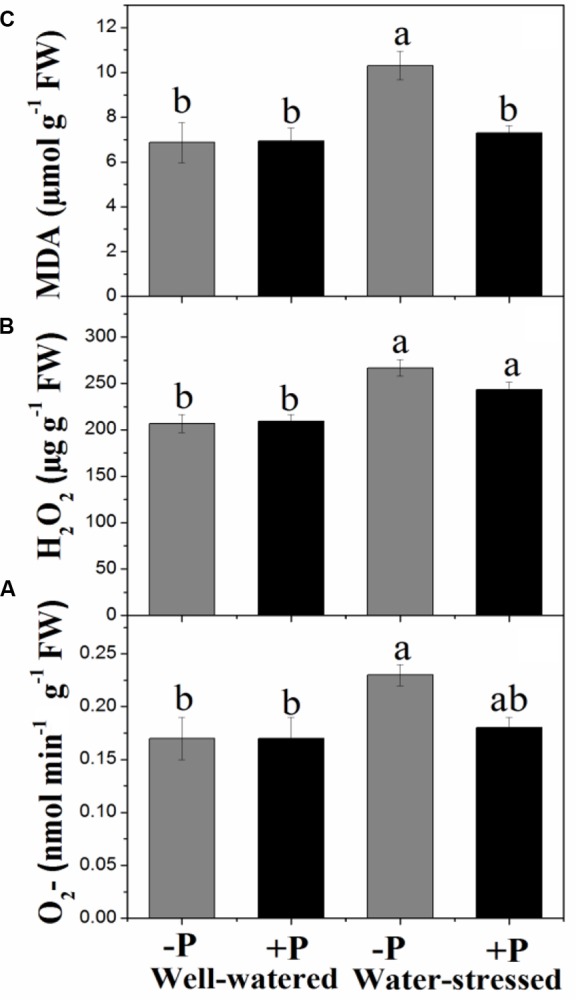
Changes in superoxide anion (O_2_^⋅-^, **A**), hydrogen peroxide (H_2_O_2,_, **B**), and lipid peroxidation (MDA, **C**) for fertilized (+P) and non-fertilized (–P) treatments with and without water. Means followed by different letters indicate significant differences (*P* ≤ 0.05) among the four treatments according to Duncan’s test. Vertical bars show ± SE.

Regardless of P application, measures of ROS production (O_2_- and H_2_O_2_) and lipid peroxidation (MDA) were significantly higher in plants under drought stress conditions than in well-watered plants (**Figure 2**). Addition of P had no significant effect on concentration of either both O_2_- and H_2_O_2_ in the two water treatments or MDA in well-watered plants; however, it resulted in significantly lower MDA content in drought-stressed plants (**Figures 2A–C**).

### Antioxidant Stress

**FIGURE 3:**
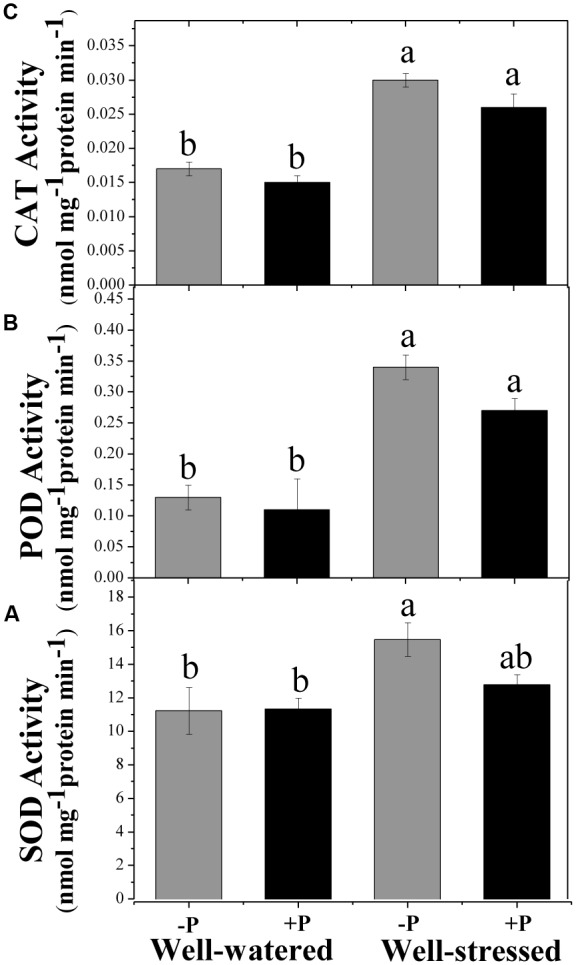
Changes in superoxide dismutase (SOD, **A**), peroxidase (POD, **B**), and catalase (CAT, **C**) for fertilized (+P) and non-fertilized (–P) treatments with and without water. Means followed by different letters indicate significant differences (*P* ≤ 0.05) among the four treatments according to Duncan’s test. Vertical bars show ± SE.

Activity of POD and CAT was higher in drought-stressed plants than in well-watered plants, and SOD activity was highest in unfertilized, drought stress plants (**Figure 3**). Addition of P had no significant effect on the measures of antioxidant stress in either of the water treatments.

## Discussion

### Drought Stress and Biomass

Drought stress is considered a major environmental stress that adversely affects tree growth and forest productivity around the world (Bartlett et al., 2012; Harfouche et al., 2014). We found that, irrespective of P application, biomass of the above and below ground plant parts of *P. zhennan* was significantly lower under drought stress conditions compared with well-watered conditions. Low water content in soil decreases mobility of available ions, nutrient availability, and microbial activities in the soil (Hu and Schmidhalter, 2001). Furthermore, root interaction with arbuscular mycorrhizas (AM) can increase plant tolerance to drought because the fungi improves plant water status by modulating ABA-mediated abiotic signaling pathway involving D-myo-inositol-3-phosphate synthase (IPS) and 14-3-3 proteins (Li et al., 2016). Moreover, in the plant it causes partial or total stomatal closure, drop in water potential, loss of cell turgor reduction of cell expansion, and if the dehydration is severe, the disruption of normal bilayer structure of the cell membranes through a reduction in synthesis, and possibly denaturation of cytosolic and organelle protein that leads to impaired cell metabolism (Mahajan and Tuteja, 2005). We found that application of P to plants under drought stress resulted in significantly greater root biomass, which may be attributed to several factors, including increased uptake of P, higher consumption rate of assimilates in root material, and enhanced hydraulic conductance of the root system (Garg et al., 2004). Phosphorous application plays an important role in root development thereby increasing accessibility to other nutrients in the rhizosphere (Liao and Yan, 2000; Razaq et al., 2017). Moreover, the root tip is responsible for sensing and signaling of P availability, and to initialize the reduction in root growth in P deficient environments and the proteins containing an SPX domain are important in regulating not only P uptake but also P distribution within subcellular compartments (Rouached et al., 2010). Higher root biomass also improves the ability of roots to extract soil moisture, contributing to drought tolerance (Meng and Yao, 2015); therefore, P application may enhance the drought tolerance of *P. zhennan* through the promotion of root biomass. This higher root biomass would be a further advantage in drought conditions where water is available in deeper soil profiles (Tardieu, 2012). We also found that P fertilization had no effect on the biomass of above and below ground plant parts in well-watered condition and suggest this may be due to the slow-growing nature of *P. zhennan*, and/or the sufficient availability of P in the soil to meet the functional requirements of the establishing seedlings. Curiously, our results contrast with studies that report increased growth rate and biomass accumulation in P fertilized plants (Graciano et al., 2005; Jones et al., 2005; Ram et al., 2006) but support other studies that suggest non-significant effects of P fertilization on growth parameters (Yin et al., 2012; Liu et al., 2015). The high variability in the responses of different plant species to P application may be because response to fertilization depend on the species, available nutrients, nutrient interactions, soil physical properties, water availability, among many other factors that modulate the response of the plant to an increase in particular nutrient concentration in the soil. Absorption of P takes place at the soil surface, and its lower diffusion rate and slower movement toward the root, compared with other nutrients, possibly affect its use efficiency (Schachtman et al., 1998; Grant et al., 2005). However, in greenhouse experiments, growing conditions are well controlled; therefore P use efficiency can be improved if P is mixed uniformly with the volume exploited by roots (Mitchell, 1957; Sample et al., 1980). However, P use efficiency varied within a species. For example in *Hordeum vulgare* the expression of the gene HVPT5, that can be used to estimate phosphate use efficiency, was higher under low P availability in a tolerant accession, but its expression did not change in the sensitive accession (Ren et al., 2016). Moreover, plants have inducible high affinity phosphate transporters and constitutive low affinity phosphate transporters encoded by *Pht* (phosphate transporter) gene family that ensure P uptake from the soil and distribution within different organelles of plant to sustain photosynthesis, respiration, and growth even under low P availability conditions (López-Arredondo et al., 2014). Member of *Pht1* gene family encoded high affinity P transporters which are mostly expressed in epidermal and outer cortex of the root cells and have already been identified as mediators of P uptake when P is limited (Schunmann et al., 2004). However, members of the *Pht2*, *Pht3*, and *Pht4* gene families were found to be associated mostly with P distribution within subcellular compartments (Versaw and Harrison, 2002; Guo et al., 2008). Moreover, recently, the role of microRNAs (miRNAs) has been revealed in the regulation of P homeostasis (Doerner, 2008). miRNA399 has been uncovered as a component of the shoot-to-root P deficiency signaling pathway, it moves via phloem and repress E2-conjugase which causes increase in the expression of root P uptake transporters and hence in the acquisition of P by the roots and its translocation and distribution to the shoot (Lin et al., 2008).

We found that leaf area of drought-stressed plants was significantly lower than in well-watered plants, irrespective of P application. Generally, water deficiency during the vegetative growth stage changes leaf turgidity and temperature, and reduces the supply of assimilates, thus inhibiting leaf growth. In drought tolerant plant species, reduction in leaf area is considered an adaptive strategy to reduce water loss through transpiration (Rostamza et al., 2011). However, the lack of difference in leaf area, but higher *P*n rate and LRWC that we observed in P fertilized, drought-stressed *P. zhennan*, suggests better acclimatory response to drought stress conditions. Dehydration tolerance of a plant can be measured using the LRWC index. In our study, LRWC in plants under drought conditions was significantly lower than that in well-watered plants. Reductions in LRWC in response to drought-stressed conditions have also been observed in different plant species (Shubhra et al., 2004; Yang and Miao, 2010). We found that P fertilizer increased LRWC in drought-stressed plants, but not in well-watered plants. The higher LRWC in fertilized, drought-stressed plants in our study may be associated with the greater biomass of P fertilized plants. Several studies have also reported improved LRWC due to either an improved ability of root to extract water or an improved conservation of water in the plant tissues (Centritto et al., 1999; Garg et al., 2004; Shubhra et al., 2004; Sato et al., 2010). However, our results contradict several studies that showed non-significant effects of P fertilization on LRWC under water deficit condition (dos Santos et al., 2004; Singh et al., 2006; Liu et al., 2015). This variability in the effect of P in drought-stressed plant species may be due to interspecific differences in physiological, biochemical, and molecular mechanisms, such as gene expression and protein assimilation. Genes induced by drought stress have been shown to not only protect plant cells from dehydration but also regulate signal transduction of certain genes, many of them encode ion transport proteins which need ATP (P rich compound) and P is also an important element in various metabolic steps of protein synthesis (Bohnert and Jensen, 1996; Bohnert and Sheveleva, 1998). Moreover, a complex intercross between P and N availability in plant water use was demonstrated in the subtropical trees *Eucalyptus grandis* and *Pinus taeda* (Faustino et al., 2013; Graciano et al., 2016; Costa et al., 2017). Differences in dry mass partitioning as well as changes in morphology and physiology in different organs explain why fertilization can affect plant drought tolerance in different direction, accordingly with the environmental factors (soil texture and moisture, nutrient availability, stress intensity and duration) and capacity of the plant to make morphological and physiological acclimations to stressful conditions.

### Gas Exchange, Chlorophyll Fluorescence and Photosynthetic Pigments

Drought stress reduces photosynthetic rate, due to a decrease in leaf expansion and associated damage to photosynthetic machinery (Wahid and Rasul, 2005). Our findings revealed that *P*_n_ significantly decreased under drought stress, but the drop was higher in unfertilized than in fertilized plants. Stomatal closure is considered to be the main factor in decreasing photosynthesis under water deficit conditions (Anjum et al., 2011). Stomatal closure in response to soil water deficit occurs because roots release high concentrations of abscisic acid (ABA) to the xylem and, as a result, the increased pH of xylem sap promotes ABA loading and subsequent transport to the shoots (Hartung et al., 2002). It is known that many drought-inducible genes respond to ABA level in leaves, for example, ABA-dependent and ABA-independent regulatory systems of gene expression can be regulated under drought stress (Zhu, 2002). Qin and Zeevart (2002) reported that protein dephosphorylation and farnesylation are responsible for ABA signaling, while Sauter et al. (2001) showed that ABA stimulates K+ ions efflux from the guard cells, resulting in loss of turgor pressure, and decrease *G*_s_. Reduction of *G*_s_ limits gas exchange, decreases *C*_i_ concentration and rates of photosynthesis, due to decline in Rubisco activity (Reddy et al., 2004). Similarly, our results indicated a significant decline in *G*_s_ and *C*_i_ under drought stress, more sharply in unfertilized than in fertilized plants. There is still debate, however, about whether drought restricts photosynthetic rate through stomatal closure or metabolic impairment (Tezara et al., 1999). Our results revealed that lower *P*_n_ was not only associated with *G*_s_ limitation, but was also due to impaired photosynthetic apparatus as reflected by significant decrease in *F*_v_*/F*_m_ during water deficit conditions. We also found that P application under drought stress conditions resulted in significantly higher *P*_n_ and *F*_v_*/F*_m_, but had no significant effect on stomatal conductance or transpiration rate. Thus, our results suggest an enhanced drought tolerance mechanism that conserves water, as indicated by increased LRWC, is stimulated by the addition of P. These results are consistent with previous studies that have reported enhanced photosynthetic activity in different plant species treated with P fertilizer under drought stress (Burman et al., 2009; Singh et al., 2013; Liu et al., 2015). In addition, other factors may have positive impacts on *P*_n_, in response to P application, such as increased production of assimilatory products (ATP and NADPH) and carboxylation activities (Lawlor and Cornic, 2002). WUE_intr_ is considered to be an important component of adaptation to drought stress and in our study; WUE_intr_ showed an opposite trend to *P*_n_, where it was significantly higher in drought-stressed plants, irrespective of P application. Therefore, plants under drought partially closed the stomata to reduce waters losses but the photosynthesis was affected proportionally in lesser extent. However, P application in our study had no significant effect on WUE_intr_ of water-stressed and well-watered plants, as has also been reported in other studies (Oliveira et al., 2014; Liu et al., 2015).

Reduction in chlorophyll concentration is a sign of oxidative stress or pigment photooxidation under drought stress (Zhang and Kirkham, 1996) and low levels of photosynthetic pigments limit the rate of photosynthesis, thus reducing primary production. Our results showed that water deficit caused significant damage to the photosystem by degrading chloroplast pigments. The degraded chloroplast pigments may have also contributed to the decreased *P*_n_ observed in our study. Similar findings have been observed in other studies that suggest drought stress damage photosynthetic pigments (Frosi et al., 2013; Rivas et al., 2013). Previous work has shown that Chl *a* and Chl *b* are susceptible to soil water deficiency (Farooq et al., 2009) Furthermore, soil dehydration has been shown to damage lamellae vesiculation and chloroplast membranes, inducing reductions in chlorophyll (Anjum et al., 2011). In our study, P application had a significant positive effect on Chl *a* and Chl *b* concentration, which may explain the higher *P*_n_ rate in P fertilized, drought-stressed plants, because high leaf chlorophyll concentration may allow for increased harvesting of light over shorter periods of time, as evidenced by the observed higher photosynthetic rates. Although there were changes in chlorophyll concentration, P fertilization did not change carotenoids concentration. This result is different of that found in the herbaceous *Petunia hybrid*, in which high P inhibited carotenoids biosynthetic genes (Nouri et al., 2015). Although our results were contradictory to some studies, in which negligible changes in chlorophyll concentrations due to P fertilization may be due to the duration and severity of drought (Zhang and Kirkham, 1996; Campbell and Sage, 2006; Singh et al., 2013). Nevertheless, our findings are supported by previous studies that suggest P application increases synthesis of photosynthetic pigments in plants under drought stress (Sharma, 1995; Sinha et al., 1995).

### Osmolytes Accumulation and Nitrogenous Compounds

Plants adapt to drought environments by increasing the solute concentration of cells to maintain osmotic function and hydration (Ramanjulu and Bartels, 2002). Plants accumulate a variety of osmolytes in the cytosol, therefore the ability to increase osmotic pressure is considered to be a potential cellular drought tolerance mechanism, as it improves or maintains turgidity and continuation of plant growth. Apart from osmotic adjustment, osmolytes also help in ROS detoxification, membrane stabilization, as well as protecting macromolecules (Keunen et al., 2013). Our results showed that there was no significant increase in SS concentration under drought in unfertilized plants. However, P fertilization significantly increased SS concentration in drought-stressed plants compared with well-watered; this may be due to the inhibition of normal SS utilization and translocation during water stress or hydrolysis of starch (Shubhra et al., 2004). Accumulation of SS protects cells in water deficit environments by substituting the hydroxyl group for water, thus maintaining a hydrophilic interaction between proteins and membranes to retain membrane integrity (Hoekstra et al., 2001). This positive effect of P fertilization on SS accumulation and mobilization clearly indicates its role in improving drought tolerance of *P. zhennan*. We found that proline concentration was significantly higher in drought-stressed plants than in well-watered. Proline accumulation in low moisture environments is due to reciprocal regulation of two pathways: up-regulation of proline synthesizing enzymes and down-regulation of proline degrading enzymes activities (Peng et al., 1996). Reddy et al. (2004) reported that the accumulation of proline in response to drought stress was regulated by a rate limiting enzyme, PFC5, in higher plants. Previous studies conducted on different plant species showed varied response of proline accumulation to P application under drought stress. For example Liu et al. (2015) suggested that P application significantly decrease proline concentration in water-stressed *Fargesia rufa*; however, Al-Karaki et al. (1996) found that P application significantly increased proline accumulation in sorghum while the bean plants showed higher accumulation at low P level than at high P level. We found that P application neither decreased nor enhanced proline accumulation in drought-stressed plants. It clearly indicates that proline accumulation responses to P fertilization in drought-stressed plants are inconsistent, varying according to specific tolerance mechanisms and level of P applied. It is already understood that proline accumulation increase under drought stress in many plant species but not necessarily with P application.

Nitrogen is an important nutrient for plant growth as it is involved in the synthesis of chlorophyll, amino acids, nucleic acid, and proteins. Generally, water stress can reduce available N uptake, resulting in a decrease in the production of nitrogenous compounds (Lawlor and Cornic, 2002; Garg et al., 2004). Similarly, our results showed lower amounts of NH_4_^+^, NO_3_^-^, and SP under limited water supply, regardless of P fertilization. Possible reasons for this decreased SP concentration in drought-stressed plants include an associated increased function of protease enzymes, proteolysis or decreased protein synthesis, as well as the lower *P*_n_, i.e., less carbon to build any metabolite. Furthermore, our findings revealed that P application slightly up-regulated NH_4_^+^ and NO_3_^-^ levels in water-stressed plants and these may be attributed to changes in associated enzyme activities (Burman et al., 2004, 2009). Azcon et al. (1996) also observed a positive effect of P fertilization on the reduction and assimilation of nitrogenous compounds. It appears that P fertilization can enhance the drought tolerance of *P. zhennan* by accumulating osmoprotectors and enhancing nitrogenous compounds reduction and assimilation.

### ROS Production, Lipid Peroxidation, and Antioxidant System

Response to drought is an inherent property of a plant, but it also depends on the length and severity of stress period. Long term drought stress causes a decline in the rate of photosynthesis, leading to an over-production of ROS (Foyer and Noctor, 2005; Carvalho, 2008). An increase in ROS level triggers protein degradation, lipid peroxidation, DNA fragmentation and may cause cell death (Apel and Hirt, 2004). ROS (O_2_^⋅-^ and H_2_O_2_) production and MDA content were found to be significantly higher in drought-stressed plants compared with the well-watered, regardless of P application because of low photosynthetic rate and other physiological disruption within the cell. Induction of antioxidant enzyme activities is a general tolerance strategy to drought stress, as it helps plants to overcome oxidative stress and associated damage. The antioxidative enzyme SOD is responsible to dismutase O_2_^⋅-^ into H_2_O_2_ in the chloroplast, mitochondrion, cytoplasm, and peroxisome, while POD and CAT play important functions in scavenging H_2_O_2_. Antioxidative (SOD, POD, and CAT) enzyme activities were also found to be significantly higher in drought-stressed plants compared with well-watered plants, irrespective of P application, suggesting a strong antioxidant defense mechanism in *P. zhennan* under drought conditions. These results indicate that antioxidative enzyme processing is substrate (ROS) inducible, leading to increased expression of genes encoding these enzymes. Reddy et al. (2004) reported that drought stress induced mRNAs corresponded to the genes of antioxidant enzymes. Similar results were also reported in other studies conducted on variety of plant species (Mittler, 2002; Murshed et al., 2013; Oliveira et al., 2014). In our study, P application resulted in slightly lower levels of O_2_^⋅-^ and H_2_O_2_ under drought stress, while level of MDA was significantly lower. P application had no significant effect on antioxidant enzymes or their activities and remained higher under drought treatment. Our findings indicate *P. zhennan* has the potential to tolerate natural drought conditions and that P fertilization may have a positive role in maintaining the tolerance capacity and mitigating the effects of drought stress.

## Conclusion

Drought stress severely affected the growth and metabolism of *P. zhennan*. However, our findings also revealed that this tree species utilizes a range of drought tolerant strategies. These strategies include decreased leaf area, limited stomatal conductance and transpiration rate, increased antioxidative activities and accumulation of osmoprotectors. P application had negligible or almost no effect on the morphological and physio-biochemical traits under well-watered conditions. However, P application had significant positive effects on the root biomass, net photosynthesis rate, chlorophyll fluorescence, leaf relative water content, and chloroplast pigment as well as in biochemical adjustments (i.e., SS) that confer improved tolerance of *P. zhennan* to drought stress. These findings provide baseline information to improve our understanding of the morphological and physio-biochemical responses of *P. zhennan* under drought stress and the positive effects of P fertilization on plants in drought-stressed environments. Balanced P fertilization may facilitate *P. zhennan* seedlings in agroforestry system if there are frequent drought events because P has positive effect on drought tolerance. We suggest further studies into underlying biochemical and molecular mechanisms under drought stress conditions, as well as and the possible role of different levels of P fertilization in mitigating negative effects of drought or for improving drought tolerance of *P. zhennan*.

## Author Contributions

KP and AT designed the study including experimental design. AT, ZL, OO, WC, and AZ carried out the physiological studies. AT analyzed the data and drafted the manuscript. CG, FS, ZL, OO, and CL helped in analyzing data, while KP, CG, XS, LZ, and CL contributed to revising the draft. DS, DM, QX, and XW contributed reagents, materials, and analysis tools.

## Conflict of Interest Statement

The authors declare that the research was conducted in the absence of any commercial or financial relationships that could be construed as a potential conflict of interest.
